# Diverse coping modes of maize in cool environment at early growth

**DOI:** 10.1186/s12870-025-06198-2

**Published:** 2025-02-13

**Authors:** Paweł Sowiński, Katarzyna Wieliczko-Manowska, Marcin Grzybowski, Maciej Jończyk, Jakub Sowiński, Alicja Sobkowiak, Piotr Kowalec, Janusz Rogacki

**Affiliations:** 1 Department of Plant Molecular Ecophysiology, Institute of Plant Experimental Biology and Biotechnology, Faculty of Biology, University of Warsaw, Miecznikowa 1, Warsaw, 02-096 Poland; 2 University of Economics and Human Sciences in Warsaw, Okopowa 59, Warsaw, 01-043 Poland; 3 Plant Breeding Smolice Co. Ltd., Smolice 146, Kobylin, 63-740 Poland

**Keywords:** Cold stress, Early growth, Low temperature, Plant phenotyping, Stress recovery, *Zea mays* L.

## Abstract

**Background:**

Maize cultivation has considerably expanded beyond its place of origin in Central America. The successful adaptation of maize to temperate climates can be achieved by selecting genotypes that demonstrate tolerance to low temperatures, especially in cold springs. In maize, cold tolerance at the early growth stages enables early sowing, a long growing season, and eventually high yields, even in temperate climates. Maize adaptation during early growth has not been thoroughly investigated; therefore, we tested the working hypothesis that several distinct and independent adaptation strategies may be involved in maize habituation to cool temperate climates during seedling establishment.

**Results:**

We studied the effect of mild cold stress (day/night 16/12 °C) on early growth stage followed by regrowth at optimal daily temperatures (24/21 °C). Automated plant phenotyping was performed on 30 inbred lines selected from a diverse genetic pool during preliminary studies. As a result, we generated time series based on selected morphological parameters, spectral parameters, and spectral vegetation indices. These curves were clustered and four classes of maize with clearly contrasting growth modes and changes in their physiological status were distinguished at low temperatures and during regrowth. Two classes comprised either cold-sensitive (slow growth and poor physiological status in cold) or cold-tolerant (moderately fast growth and good physiological status in cold) lines. However, two other classes showed that growth rate and physiological status at low temperature is not necessarily related, for instance one class included lines with small seedlings but good physiological status and the other grouped seedlings with rapid growth despite poor physiological status. These classes clearly exhibited different modes of cold adaptation. Moreover, a class containing cold-sensitive inbred lines may represent a distinct and novel type of cold-adaptation strategy related to the arrest of coleoptile emerge related with ability to recover rapidly under favourable conditions.

**Conclusions:**

Our results support the hypothesis that maize may have several adaptation strategies to cold environments at early growth stages based on independent mechanisms. These findings suggest that maize adaptability to adverse environments is likely more complex than previously understood.

**Supplementary Information:**

The online version contains supplementary material available at 10.1186/s12870-025-06198-2.

## Background

The cold sensitivity of maize is a major concern for agriculture in many regions of Eurasia and America, and the response of maize to cold conditions has been studied for decades. In the field, maize can be affected by low temperatures mainly at the beginning of its vegetation, i.e., when coleoptile emerges from the soil (VE growth stage, growth stages according to https://crops.extension.iastate.edu/encyclopedia/corn-growth-stages) following by developing of the juvenile leaves (V1–V7 growth stages, V(n) – full development of the n^th^ leaf). This relatively short period of vegetation consists of two phases being affected by cold in quite different ways. Initial growth and development, the VE–V2 growth stages, depends mainly on seed reserves while the photosynthetic apparatus is being formed; at approximately 13 °C, seedlings of cold-sensitive inbred lines may die when the seed reserves are exhausted before the seedling reaches the autotrophic stage [1, 2]. In contrast, V3–V7 seedlings do not suffer serious damage at moderately low temperatures (12 °C–15 °C), only severe cold, below 6 °C, can cause membrane injury, bleaching, necrosis, or even death at this stage [3]. Thus, the transition from heterotrophic to autotrophic growth is cold-sensitive stage.

Stress avoidance and tolerance are the two main mechanisms that enable plants to adapt to harmful environments [4]. In the VE–V3 stages, avoidance includes xeromorphic seedling shoot architecture expressing in maize as short and chunky plants. This growth behaviour prevents water loss under cold conditions [5–8]. Another example is inbred lines of maize in which the shoot apex remains protected in the soil during the transition from heterotrophic to autotrophic growth, facilitating optimal development of new leaves and chloroplasts at low temperatures [8]. Such mechanisms are complex and qualitative and cannot be identified or characterised by analysing a single parameter. In contrast, the true cold tolerance is a quantitative trait. Nevertheless, true cold tolerance can be discrete, manifesting when a combination of parameters, rather than a single factor, is considered. This is supported by the clustering analysis of Enders et al. [9], which identified several distinct classes of maize responses to severe cold. Therefore, it is reasonable to speculate that the adaptation of maize to cool spring conditions may exhibit multiple characteristics, reflecting various strategies that facilitate habituation to a temperate climate. Such adaptation strategies could be distinguished by markedly contrasting growth modes and modifications in physiological status at low temperatures and during regrowth. This concept is particularly likely in the case of maize, a species with exceptional genetic and phenotypic diversity.

Three-dimensional automated plant phenotyping presents an approach that would aid the study of different plant adaptation strategies to adverse conditions [10]. This approach, based on the application of non-invasive digital imaging technologies, is a modern technique that has been increasingly used in plant physiology. According to many authors, 3D automated plant phenotyping is at the forefront of future plant breeding [11], as it provides data on phenotype that determine the utility values of the new cultivars. A particular application of the automated phenotyping is the study of the plant stress responses [12]. This approach provides a comprehensive overview of plant disease symptoms based on colour analysis sometimes before other signs of stress. Certain solutions also offer 3D images of plant morphology, which could be used to calculate many morphological traits, such as shoot height and 3D leaf area or leaf inclination [10]. Finally, the 3D images could be taken in time series over weeks or months, facilitating the study of the dynamics of longer-term changes. The obtained time series of several heterogeneous parameters need to be processed and analysed to provide a comprehensive understanding of plant development and its health status; this can be accomplished through the clustering of these multi-dimensional time series datasets [13]. In general, clustering is the separation of the data into different groups based on their similarities. Clustering can also be used to analyse time profiles. In plant physiology, hierarchical clustering of time profiles has already been successfully used to qualify the response of maize to severe stress and post-stress recovery [9]. Another type of time series clustering, specifically aimed at analysing multi-dimensional profiles is k-mean clustering, [13] which is mainly used in human health [14] or industry [15]. However, as the purpose of this approach was data mining of multi-dimensional time series, it is reasonable to assume that this tool would be suitable for analysing of 3D image time series resulting of plant phenotyping.

In search of different adaptation strategies in maize at early growth stages, in this study, we examined three aspects of plant performance—growth, photosynthetic activity and physiological status at low temperatures to detect possible diversity. As cold tolerance may be attributed to plant performance at low temperatures, as well as its rapid recovery after the onset of favourable conditions, we monitored the relevant indicators both during cold treatment and during plant regrowth at optimal temperatures using automated plant phenotyping.

## Methods

### Plant material and overall study layout

**Fig. 1:**
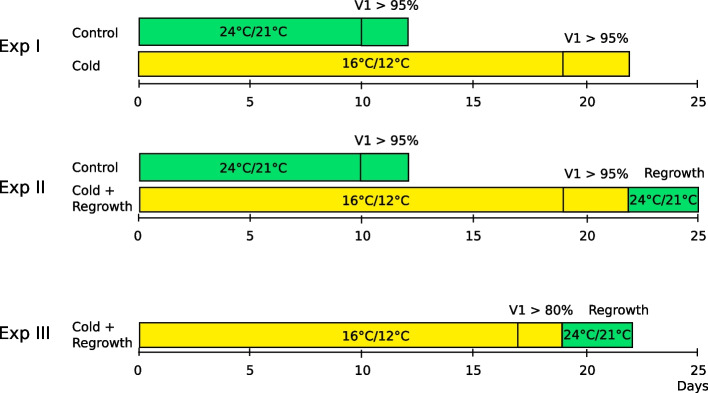
A summary of the experimental details of the project. Exp I: 64 inbred lines were used in Experiment I. Seedlings were grown to the V1 growth stage (first leaf developed in 95% of plants) at 16/12 °C for 19–22 days (yellow rectangle) or at 24/21 °C for 10–12 days (green rectangle). Measurements of Fv/Fm and PRI were taken on the first leaf using handheld instruments. Exp II: The plant material used in Experiment II consisted of 23 inbred lines selected in Exp I. Seedlings were grown till the V1 growth stage (first leaf developed) was completed in 95% plants, i.e. either for 19–22 days at 16/12 °C (yellow rectangle) and then grown under cold conditions for 3 days at 24/21 °C (regrowth, green rectangle). In addition, seedlings were grown to V1 growth stage under control conditions, i.e. at 24/21 °C for 10–12 days (green rectangle). Measurements with the 3D scanner (3D leaf area, digital biomass, leaf hue, Green Leaf Index, NDVI, NPCI, and PSRI) were taken at V1 stage between 11:00 and 13:00 for both cold grown plants and control plants, as well as for plants after 3 days of regrowth. Exp III: The plant material used in Experiment III consisted of 30 inbred lines, 21 of which studied in EXP II and 9 elite inbred lines. Seedlings were grown at 16/12 °C (yellow rectangle) until at least 80% of plants had reached the V1 growth stage (first leaf developed), i.e. 17–19 days. Plant growth under cold was followed by 3 days of regrowth at 24/21 °C (green rectangle). Measurements with the 3D scanner (except those listed above these: height, height max, leaf angle, area index, leaf inclination, leaf penetration depth, and projected leaf area) were taken continuously. The time series used for clustering concerned the last 7 days of growth at low temperature, followed by 3 days of regrowth

Maize (*Zea mays* L.) seedlings in the V1 growth stage (lowest leaf has a visible collar; this leaf has a rounded tip; growth stages according to https://crops.extension.iastate.edu/encyclopedia/corn-growth-stages.) were studied over three rounds (Fig. 1). In Experiment I, 64 inbred lines (Additional file 6) were analysed for the performance of the photosynthetic apparatus and light reflectance of the first leaf of seedlings grown at optimal or cold temperatures. In Experiment II, 23 inbred lines selected from the first experiment (Additional file 7) were analysed from morphological parameters, leaf colour, and several spectral vegetation indices in seedlings grown at low temperatures as on the third day of recovery at optimal temperatures. In Experiment III, 30 inbred lines (Additional file 8), were studied for the same parameters as in Experiment II, but in a continuous manner from the VE growth stage (coleoptile has emerged from the soil) to the V1 stage at low temperatures, followed by 3 days of recovery.

### Growth conditions

Plants were grown in a custom-made phytotron under a 14/10 h photoperiod, light irradiance of 500 μmol quanta m^−2^ s^−1^ (LED sources: BX180, light spectrum type: NS1, VALOYA, Finland), 60% relative humidity (RH), and temperature as follows: optimal (control) and recovery conditions at 24/21 °C (day/night) and low-temperature growth at 16/12 °C.

The kernels were sown in soil at a depth of 4 cm. In Experiment I, plants were grown in perforated-bottom PVC pots (6 cm × 6 cm × 6 cm), with four plants per pot, and placed in a container (2.0 × 0.6 m) filled with the same soil. In Experiments II and III, plants were grown in PVC tubes 30 cm height and a diameter of 7.5 cm, with one plant per tube.

### Measurements

#### Experiment I

Measurements were collected at room temperature (21–24 °C) for both control and cold-grown plants. Before the measurements, the pots were transferred to a dark room for 30 min. Photosynthetic activity was assessed based on the OJIP test using a PAR-FluorPen FP110 portable device (Photon System Instruments, Czech Republic). The leaf reflectance-based indices were determined using a portable CCI-710 Miniature Leaf Spectrometer (ID Bio-Science Inc., USA). Plants were grown for 19–22 days at low temperatures (16/12 °C) till 90%–95% of seedlings had reached V1 growth stage (Fig. 1). Control plants grown at 24/21 °C reached the V1 growth stage (90%–95%) 10–12 days after sowing. Measurements were performed on the middle part of the fully developed first leaf (V1 growth stage) between 10:00 and 14:00, with three or four plants per line. The experiment was repeated thrice.

#### Experiment II

Plant phenotyping was performed using a multispectral three-dimensional (3D) scanner (PlantEye F500; Phenospex, The Netherlands) with the scanning area 190 cm length and 95 cm wide. The scanner continuously captured images of the plants; however, we only used data taken between 11:00 and 13:00 at the end of either at low temperature (16/12 °C) or end of 3-day- long regrowth (24/21 °C). Among several parameters, we considered two morphological parameters, 3D leaf area [mm^2^] and digital biomass (product of plant height and 3D leaf area) [mm^3^]; two spectral parameters, leaf hue (colour analysis of plant images, alternative to RGB) and the Green Leaf Index (the reflectance in the green channel vs that in red and blue); and three spectral vegetation indices, normalised difference vegetation index (NDVI), normalised pigment chlorophyll ratio index (NPCI), and plant senescence reflectance index (PSRI).

The inbred lines were divided into two sets according to their rate of development to the V1 stage (fast- and slow-growing) to obtain seedlings at the same stage of development before recovery began. The plants of different inbred lines were randomly distributed along the scanning area. Plants were grown for 19–22 days at low temperatures until 90%–95% of seedlings reached the V1 growth stage (Fig. 1). Then temperature was changed to optimal ones for recovery. Scanning was activated when the coleoptiles started to emerge above the soil surface (VE growth stage), i.e., 9–11 days after sowing. The control plants reached V1 growth stage (90%–95%) 10–12 days after sowing and the scanner was activated 4–5 days after sowing. The experiment was repeated three to five times with five plants per line per experiment.

#### Experiment III

Automated plant phenotyping was performed continuously (one scan every 20 min) by using the aforementioned scanner. In addition to the parameters mentioned above, several other morphological parameters were measured during automated phenotyping. These were height (the top 10% of the plant is averaged, and the height is calculated as the range from pot height up to this averaged value), height max (the absolute highest point of the plant), leaf angle (value range: 0–90°, the weighted average of all angles of every face in the plant mesh based on their normal), 3D leaf area (value range: 0–∞), leaf area index (value range: 0–∞, [3D leaf area/sector size]), leaf inclination (value range: 1–∞, [total leaf area]/[projected leaf area]), leaf penetration depth (the depth of the laser light penetration through the canopy of the plant), and projected leaf area (measures the area of the projection of the plant onto the X–Y-plane and turns the 3D object into a flat 2D object).

The inbred lines were divided into three groups according to their rate of development at low temperature (fast-, medium-, and slow-growing plants) up to the V1 stage to obtain seedlings at the same stage of development before the start of recovery after the onset of optimal growth temperature. The inbred lines were selected from preliminary studies (Experiment II). The experimental set was supplemented with several elite inbred lines from the United States and Europe. Each group comprised 10 lines. Pots containing plants were placed in the scanning area in five rows (19 cm wide) and 20 columns (9.5 cm wide), resulting in 100 scanning positions (each inbred line was represented by 10 plants). To minimise temperature and light gradients, four additional rows of pots with plants were positioned outside the scanning area (two rows on each side). The pot positions were determined randomly, separately for each replicate. To avoid grouping plants of a single inbred line side by side, a single draw included 10 lines of a given group, the draw was repeated 10 times, and the resulting sets of lines were arranged in rows sequentially.

Plants were grown for 17–19 days at low temperatures (16/12 °C) until at least 80% of seedlings had reached V1 growth stage. Then temperature was changed to optimal (24/21 °C). Scanning was activated when the coleoptiles started to emerge above the soil surface (VE growth stage), i.e., 9–11 days after sowing. The experiment was repeated thrice (three inbred line groups with three repetitions each).

### Data management and statistics

Statistical analyses of the data obtained from Experiments I and II were performed using R software (v.4.1.2 [16]). The emmeans function (also known as lsmeans; [17]) was used to perform posthoc pairwise comparisons between all inbred lines separately for each treatment condition. The Tukey’s method was used to adjust *P*-value with ɑ = 5%. Principle component analysis (PCA) was performed using the PCA function of the FactoMineR package with default settings, and raw data were scaled to unit variance [18].

Phenotyping data from the scanner obtained in Experiment III were saved in the *. csv format, Clustering of time series of morphological, spectral parameters, and spectral indices was performed using the K-Multi-Dimensional Time-Series Clustering Algorithm (K-MDTSC) [13]. K-means clustering method is non-hierarchical algorithm, so the user must specify the number of clusters in advance. The algorithm moves objects from cluster to cluster until the variability within clusters and between clusters is optimised.

Due to diurnal fluctuations of spectral indices (Fig. 7), only data obtained between 11:00 and 13:00 were used to generate the time series for clustering. The time series pertains to 7 days before temperature shift in the growth chamber (low temperature) and following 3 days (regrowth). The code for executing the clustering algorithm was adapted from the SmartData@PoliTO Centre repository (https://github.com/smartdatapolito/K-MDTSC). The number of classes (four) and the number of iterations (100) were selected arbitrarily. To normalise and standardise the raw data, we applied a z-transformation with the mean calculated for all experimental data. The means for all parameters are presented in Additional file 11.

## Results

### Diverse photosynthetic performance and leaf reflectance after cold stress in maize inbred lines

The inbred lines studied in the first experiment included lines tested earlier for cold tolerance [2, 8, 9, 19–21] and several elite lines. The maximal quantum yield of PSII (Fv/Fm) and the photochemical reflection index (PRI) gauging non-photochemical quenching, and photosynthetic capacity under stress and recovery [22] are shown in Additional file 1 and results of statistical analyses are presented in Additional file 10. The control values of Fv/Fm were almost identical for all the lines and varied between 0.7–0.8 (Additional file 1), which is typical for maize inbred lines. In contrast, the Fv/Fm values determined for the cold-grown plants varied considerably among the lines: in the first quartile, Fv/Fm ranged between 0.12–0.4, and in the fourth quartile, between 0.56–0.66. The PRI was highly variable in control and cold-grown plants, and the extent of its response to cold varied considerably (Additional file 1), which complicated drawing robust conclusions. Nevertheless, the PRI and Fv/Fm were significantly correlated in the control (*r* = 0.2773, *p* < 0.05) and cold-grown plants (*r* = 0.3485, *p* < 0.01).

### Regrowth processes during the recovery versus physiological status of maize cold-grown seedlings

To conduct a comprehensive study, we selected 23 lines representing the entire range of Fv/Fm values in Experiment I (Additional file 1), particularly those with low PSII activity at low temperatures. Quartiles 1–4 were represented by seven (quartile 1), seven (quartile 2), five (quartile 3), and four (quartile 4) inbred lines, respectively. Experiment II was based on three-dimensional (3D) plant scanning performed at the V1 growth stage (control and cold-treated seedlings) and after 3 days of regrowth at the optimal temperature (for cold-treated plants).

**Fig. 2:**
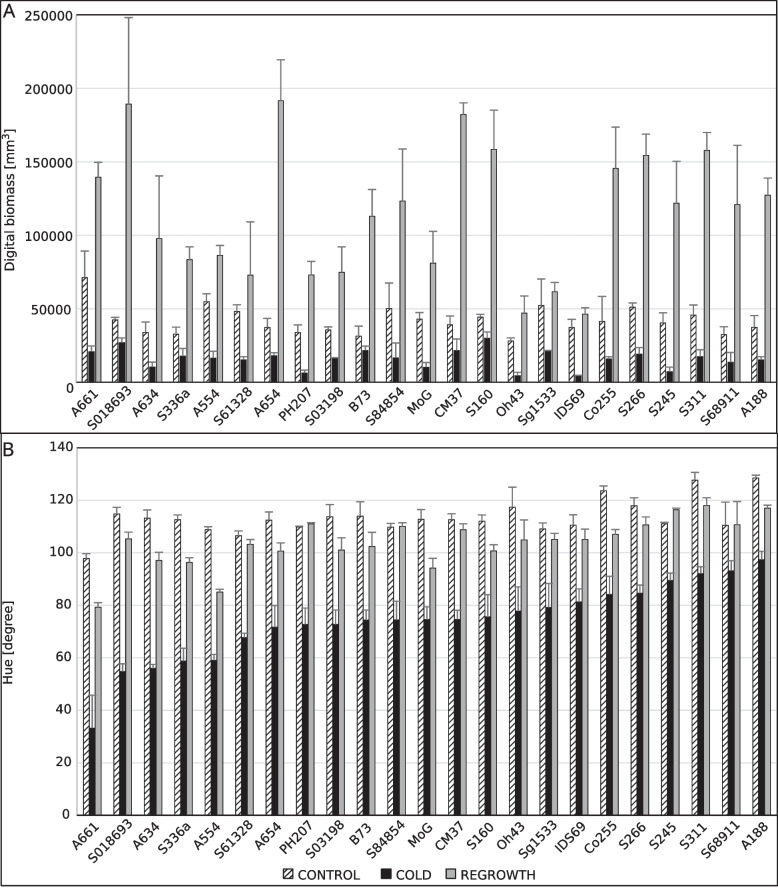
Digital biomass and leaf hue of maize seedlings at the V1 growth stage. Measurements were performed on plants of 23 inbred lines grown at optimal temperatures (24/21°; CONTROL) or at low temperatures (16/12 °C; COLD) and after 3-day-long recovery at optimal temperatures (REGROWTH). Results of statistical analysis is shown in Additional file 10. Inbred lines were sorted according to their hue values for plants grown at low temperatures. Experiments were repeated three to five times, with five plants per line per experiment. **A** – Digital biomass [mm.^3^]; **B** – Hue [degree]

The inbred lines showed variations in plant growth characteristics (digital biomass) (Fig. 2a) in both control and cold-treated plants. However, the biggest diversity was found in respect to improvement of digital biomass during 3 days of regrowth. In some inbred lines the ratios of digital biomass at the end of regrowth and at low temperatures were higher than ten times.

Leaf hue reflects chlorophyll content and is indicative of photochemical yield [23]. To present leaf colours in the text in an objective manner, regardless of the monitor or printer used, we provide the hexadecimal codes for each colour. They were obtained for a given hue value by means of colour converter (e.g. https://www.w3schools.com/colors/ colors_converter.asp) by setting H(ue) in degrees and the arbitrarily selected values of S(aturation) and L(ightness) of 100% and 50%, respectively. Then other on-line tool was used (e.g. Html Css Color; https://www.htmlcsscolor.com/) to obtain the colour name. Among the lines tested, the extreme cases were lines A661 and S018693 (Fig. 2b), with orange (#ff8c00 code) leaves, and line A188, with bright green (#5eff00 code) leaves. Other lines represented a spectrum of intermediate colours from yellow (#ffff00 code) to chartreuse (#80ff00 code). Leaves changed colour significantly during regrowth in all lines, with the most pronounced changes in lines S018693, which became bright green (#55ff00 code), and S311 which colour turned to lime (#00ff00 code). However, the restoring of leaf colour was not fully effective, and it only reached control levels in several inbred lines.

The other parameters and indices concerning only plants developed under cold conditions and after the regrowth period are shown in the Additional Material (Additional file 2 and 3).

**Fig. 3:**
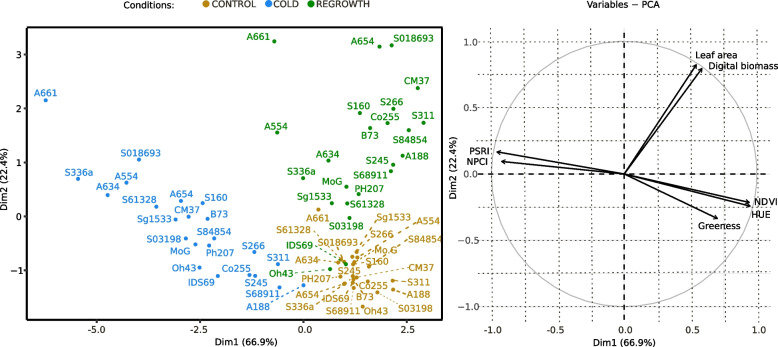
Principal component analysis (PCA) of data obtained for seedlings of 23 inbred lines grown at optimal temperatures (24/21 °C; CONTROL) or at low temperatures (16/12 °C; COLD) and after 3-day-long recovery at optimal temperatures (REGROWTH). PCA was performed using the PCA function of the FactoMineR package with default settings, with the raw data scaled to unit variance [18]

To identify a pattern in the variable performance of the inbred lines, we performed a PCA (Fig. 3). Spectral parameters and spectral vegetation indices strongly affected PC1, whereas morphological parameters influenced PC2. Thus, the PCA showed the lack of correlation between morphological parameters as well as spectral parameters and spectral vegetation indices. Furthermore, regrowth processes during the recovery period and the physiological status of cold-grown plants are independent factors.

### Diverse modes of maize growth at low temperatures and regrowth after cold-stress exposure

Thirty inbred lines (Additional file 8) were used in this experiment, including the 21 inbred lines studied in Experiment II (two inbred lines from the original set, PH207 and S61328, were excluded) and nine elite inbred lines. To classify the inbred lines into distinct groups differing in physiological status and growth rate under cold conditions and during regrowth, we clustered the obtained time series based on selected morphological parameters, spectral parameters, and spectral vegetation indices (see Materials and methods).

**Fig. 4:**
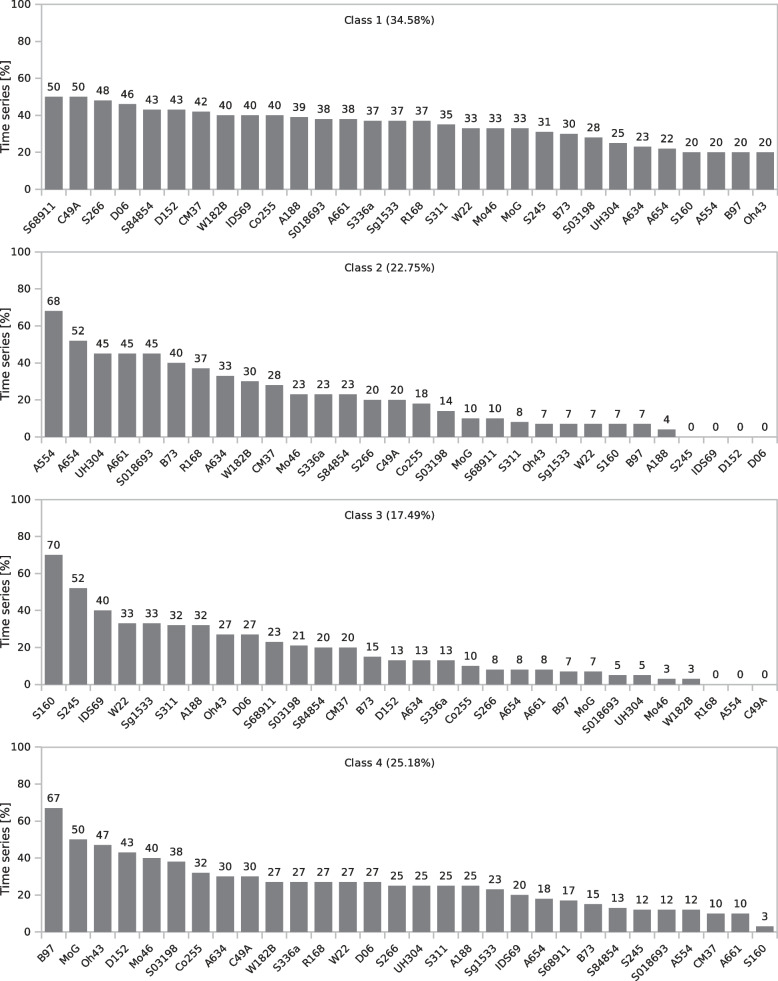
Percentage of 30 inbred lines in four classes obtained by clustering of the time series of changes in morphological parameters, spectral parameters, and spectral indices at low temperatures (16/12 °C; COLD) until V1 growth stage and after 3 days of regrowth at optimal temperatures (REGROWTH). Number of classes was chosen arbitrarily. The bars represent the percentage of time series for a given inbred line assigned to a particular class. The sum of the percentages for a given inbred line in all classes is 100%. Time series clustering was performed using the K-Multi-Dimensional Time-Series Clustering Algorithm (K-MDTSC) [13]

**Fig. 5:**
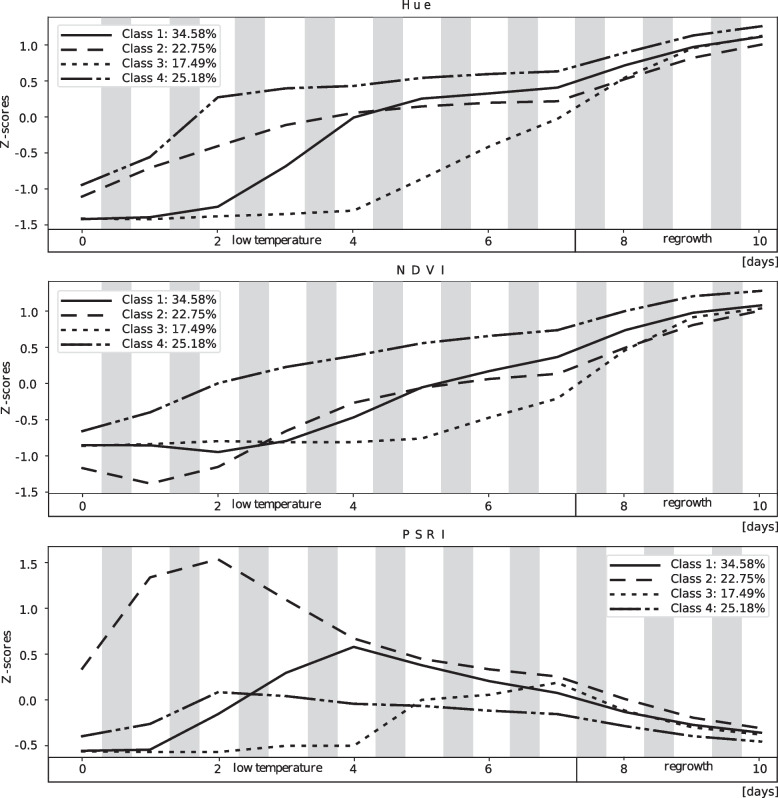
Averaged time series of spectral parameter hue and the spectral vegetation indices NDVI and PSRI for the four classes shown in Fig. 4. Data were subjected to a z-transformation, with mean and standard deviation calculated for all experimental data (data shown in Additional file 11). The time series pertain to 7 days before temperature shift in the growth chamber (low temperature) and the following 3 days (regrowth). White and grey stripes mark day (14 h) and night (10 h) periods, respectively

**Fig. 6:**
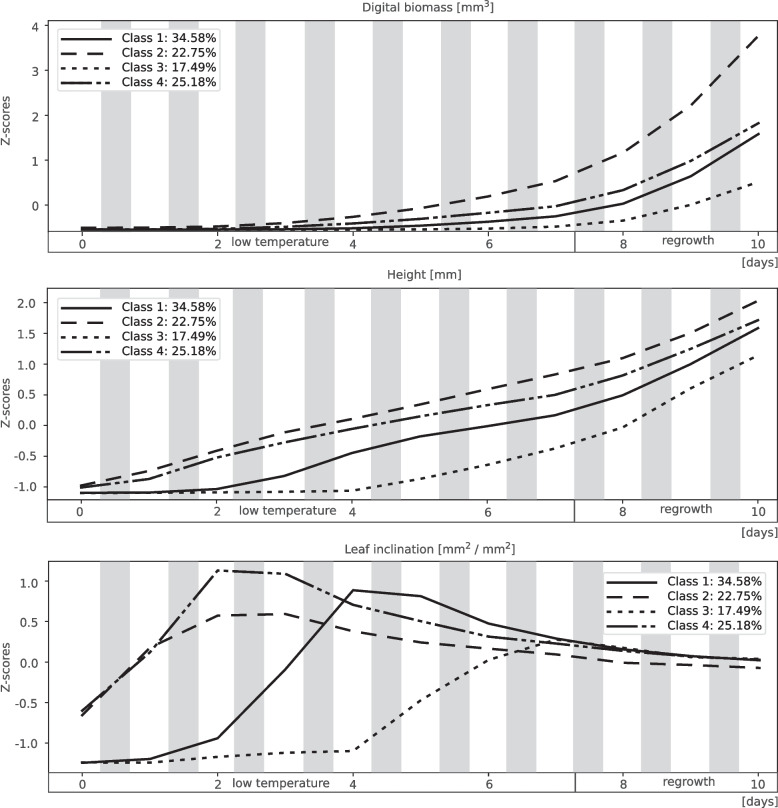
Averaged time series of morphological parameters digital biomass, height, and leaf inclination for the four classes shown in Fig. 3. Other descriptions as on Fig. 5

**Fig. 7:**
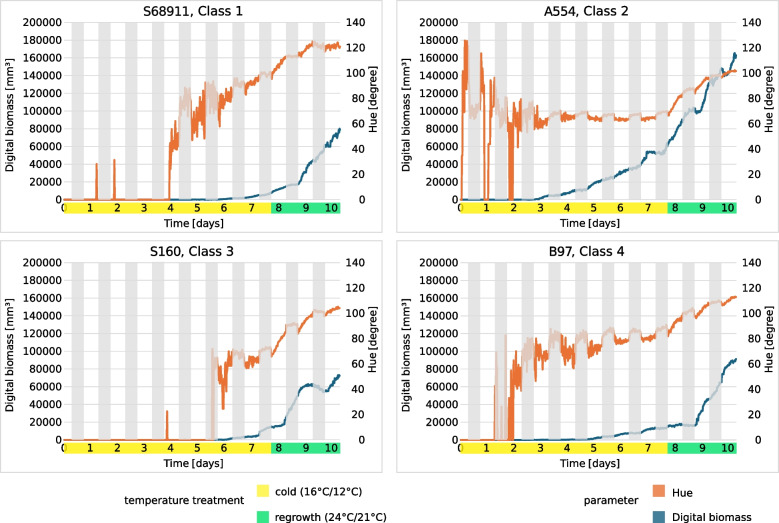
Examples of single time series of digital biomass and HUE from inbred lines most representative of the classes shown in Fig. 4: S68911, Class 1; A554, Class 2; S160, Class 3 and B97, Class 4. White and grey stripes mark day (14 h) and night (10 h) periods, respectively. Cyclic changes in HUE are the effect of photoperiod and are typical for time series of spectral indices

**Fig. 8:**
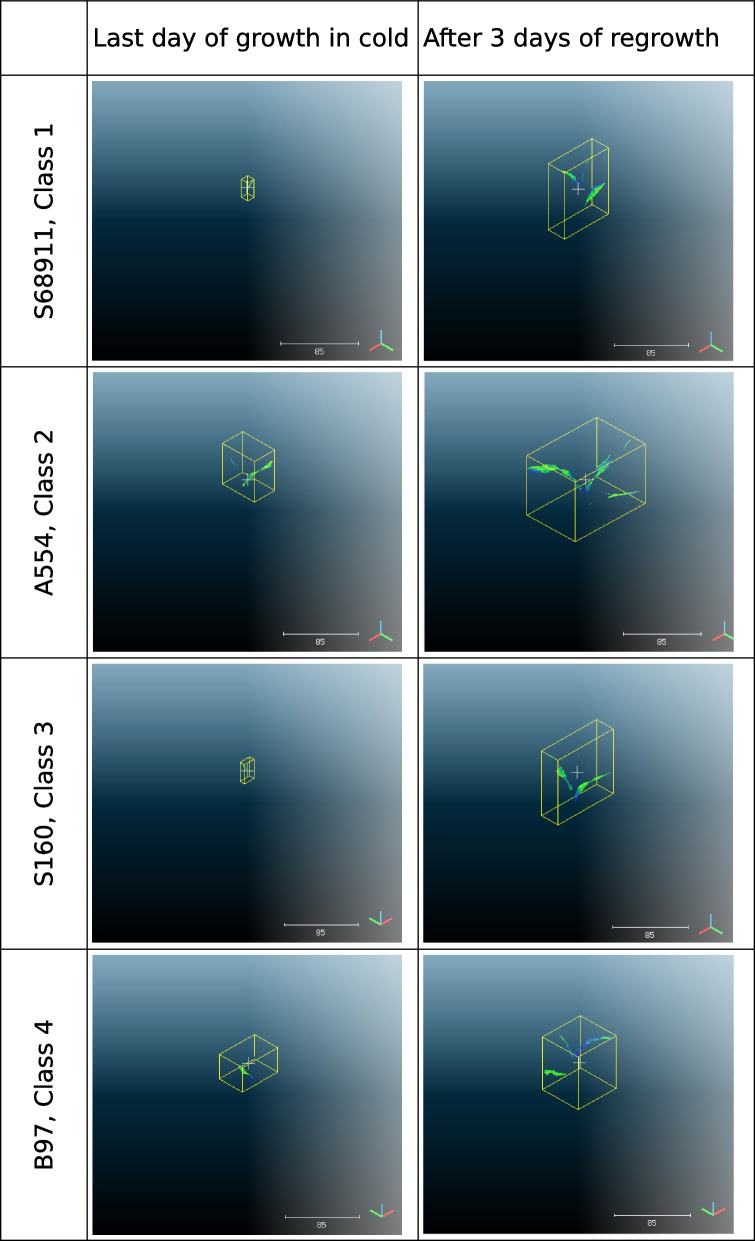
Examples of single scans of plants from inbred lines most representative of the classes shown in Fig. 4: S68911, Class 1; A554, Class 2; S160, Class 3 and B97, Class 4. Scans in columns refer to a single inbred line. Scans of seedlings at V1 growth stage on the last day of growth at low temperature and after three days of regrowth at optimum temperature are shown in the top and bottom rows, respectively. The scans have been prepared for publication using of CloudCompare software. The scans have not been edited except for cropping

The four classes representing the clustering effect are shown in Fig. 4. The number of classes and the number of iterations were chosen arbitrarily. The bars represent the percentage of time series for a given inbred line assigned to a particular class. The sum of the percentages for each inbred line in all classes was 100%. Figures 5 and 6 present the average time series for the four classes. The data were z-transformed (see Materials and methods). Means and standard deviation of all parameters are presented in Additional file 11. Along the body text, only digital biomass, leaf area, leaf inclination, leaf hue, NDVI, and PSRI are shown. The other parameters and indices are presented in the Additional Material (Additional file 4 and 5). In addition to the clustering result, examples of both individual time series and scans of representative inbred lines of all four classes are shown in Figs. 7 and 8.

Classes 1, 2, 3, and 4 represented 34.58%, 22.75%, 17.49%, and 25.18% of the time series, respectively. Notably, classes 2, 3, and 4 each contained two inbred lines characterised by a high proportion (> 50%) of time series typical of a given class and a few by a medium proportion (> 40%). The remainder was distributed among different classes, especially class 1.

Class 2 had the fastest growth, evidenced by the large increase in digital biomass over time (Fig. 6; numerical data for the mean and standard deviation are shown in Additional file 11); however, the plants in this class were chlorotic, rather unhealthy, and showed clear signs of stress (mean values on the last day of cold growth: hue = 64.26° [HEX colour #eeff00 = chartreuse yellow], NDVI = 0.1840, and PSRI = 0.4190) (Fig. 5). In contrast, class 4 showed the best physiological status of the cold-grown and recovering seedlings (hue = 71.94° [HEX colour #ccff00 = Electric lime], NDVI = 0.2011, PSRI = 0.3020) (Fig. 5); however, growth was moderate(Fig. 6). A specific feature of class 3 was the notably slow growth at low temperatures (Fig. 6), delayed by several days compared with class 1, accompanied by a delayed increase in the values of the spectral indices (Fig. 5), but strongly accelerated during the regrowth period. Class 1 was intermediate, between classes 3 and 4. Similar to class 3, its physiological status was low at the beginning of growth at low temperatures but improved rapidly. In contrast, growth in class 1 was slow, similar to that of class 4. Notably, another similarity was evident between classes 1 and 4, namely, a rather high value of leaf inclination, which indicates high leaf erectness.

## Discussion

We compared various aspects of seedling growth and the physiological status of dozens of inbred lines to test whether the ability of maize to develop at suboptimal temperatures is diverse. Accordingly, we expected to identify several groups of inbred lines with different growth dynamics and/or changes in physiological characteristics at low temperatures and during subsequent regrowth. To this end, we used a combination of classical and new approaches, i.e., assessment of plant conditions with handheld analysers at selected times and continuous monitoring of plant growth and physiological status with automated plant phenotyping.

In a preliminary study, we evaluated a set of 64 inbred lines selected based on our earlier research and the literature [2, 8, 19–21] (Additional file 1). The aim of this step was to select a defined but manageable set of inbred lines representing a broad spectrum of photosynthetic activities under chilling conditions during early growth. This stage of maize development is considered critical for photosynthetic apparatus formation and, thus, for the survival of maize seedlings under cold conditions [1, 8]. The material was tested using Fv/Fm and PRI for the control and cold-treated plants. The first index reflects the maximum quantum yield of photosystem II photochemistry [24] and is based on chlorophyll fluorescence. The second index, which is based on leaf reflectance, is related to non-photochemical quenching and photosynthetic capacity under stress and recovery [22]. Fv/Fm was used for the initial selection of material for further studies, whereas the final selection of 23 inbred lines was performed using the PRI index.

The selected set of inbred lines was then analysed for several morphological characteristics and spectral indices using a 3D multispectral scanner at the V1 growth stage (control, cold-grown seedlings) and after 3 days of regrowth at the optimal temperature (regrowth) (Fig. 2). According to Lainé et al. [25], the recovery after the severe cold spells were not related to growth during cold. It could be therefore hypothesised, that the ability to recover in maize seedlings may be a separate part of the cold-adaptation strategy. The aim of this part of the study was to test whether plant recovery depended on the state achieved at moderately low temperatures. According to the PCA (Fig. 3), the ability of seedlings to recover at optimal temperatures is only slightly dependent on their physiological status at the end of low-temperature growth and is, therefore, a specific feature of a given inbred line. To investigate this further, we used a continuous automated phenotyping approach covering both the period of growth in cold and regrowth after the onset of favourable temperatures.

Automated phenotyping is often used to monitor plant growth in response to various stressors [9, 26–28]. We used this method to test the hypothesis of the manifold character of maize response to suboptimal temperatures at the early growth stages possibly indicating diversified adaptation strategies to cope with cool spring conditions. Through continuous monitoring the growth and physiological status of the seedlings of 30 inbred lines and clustering the time series of changes in morphological, spectral parameters, and spectral indices, we distinguished four classes of inbred lines that differed in the dynamics of changes in growth and physiological characteristics at low temperatures and during subsequent regrowth (Fig. 4).

The best physiological status of seedlings under cold conditions (high leaf hue and NDVI; low PSRI) was represented by class 4 (Fig. 5), which is also characterised by moderate growth at low temperatures and during regrowth (Fig. 6). Another specificity of class 4 was the high values of the leaf inclination trait and, consequently, high light interception by the leaves. Leaf inclination is a plant trait that maximises yield [29]. However, this relationship has been described for adult plants and the relationship between leaf inclination and plant performance at the seedling stage at low temperatures remains unclear. Several cold- or moderately cold-tolerant inbred lines belong to class 4. These include the inbred line B97, which is considered to be well adapted to temperate regions [30], as well as D152 [20, 31], S03198 [8], and Co255 [2, 32]. Maize tests under severe cold conditions showed cold tolerance in A634 [21] and Oh43 [9, 21] but cold sensitivity in the inbred line MoG [21]. Therefore, class 4 represents inbred lines that are well adapted to cold environments but possibly with a predominance of moderately low temperatures.

The opposite of class 4 was class 2, which represented the fastest growth of seedlings at low temperatures and during regrowth (Fig. 6), with a poor physiological status (chlorotic leaves, low NDVI, and high PSRI at the beginning of first-leaf development stage) (Fig. 5). Similar mechanism was mentioned by Strigens et al. [7] in inbred lines belonging to the European dent (EU-D) breeding group. This cold adaptation mechanism was based on efficient biomass accumulation despite low photosynthetic capacity. Apparently, the aforementioned adaptation mechanism is not limited to EU-D breeding groups, as two representatives of class 2, A554 and A654, are elite US inbred lines (Additional file 8), whereas S018693 is a flint line. To our knowledge, the cold response of A554 and A654 has not been studied before. Other representatives of class 2, line A661 was found to have an albino phenotype at low temperatures but with a rapid recovery of chlorophyll content at optimal temperatures [19], whereas S018693 and UH304 were described as moderately cold tolerant [8, 20]. Notably, the rapid growth of these lines at low temperatures was accompanied by the development of new leaves; four leaves were visible at the V1 stage (data not shown). To summarise, class 2 represents inbred lines well adapted to cold environments, however the underlying mechanism is apparently different than in class 4.

Class 1 shows growth dynamics similar to those of class 4 (Fig. 6) and a good physiological status at low temperatures (Fig. 5). Class 1 also expressed high values of leaf inclination, similar to class 4, but only during the second half of the growth period when exposed to low temperatures. This might reflect the slow growth of plants exposed to low temperatures. Notably, visual assessment of seedling germination showed that the VE growth stage of class 1 was delayed by 2–3 days compared with that of class 2 (data not shown). Class 1 includes several cold-tolerant and moderately cold-tolerant inbred lines tested after severe cold stress, including C49A, D06, IDS69, and Sg1533 [9, 21]. Class 1 also includes two cold-tolerant inbred lines studied at moderately low temperatures (S68911 and S84854), which have been described as xeromorphic-type seedlings [8], i.e. short and chunky plants. This body shape may protect plants from water loss and secondary water stress under cold conditions [7]. Class 1 was characterised by rapid development (3–4 leaves visible at the V1 growth stage, data not shown), despite low values for all morphological characteristics. Therefore, the xeromorphic structure of seedlings can be assumed as the dominant body shape in this class.

Class 3 differs from the other three classes in its morphological characteristics and physiological status. In particular, plants in this class show slow growth and development in the cold (Fig. 6). The VE stage in this class was delayed by 3–4 days as compared to other ones. Physiological characteristics such as leaf hue and NDVI (Fig. 5) were also low but only during the first half of the growth period at low temperatures. In the following days, a rapid improvement in these characteristics was observed, especially during regrowth, when their values reached the level of that of classes 1 and 2. The main feature of class 3 plants is the cessation of processes related to germination and/or elevation of the coleoptile above the ground at low temperatures. Representatives of class 3 are the inbred lines S245 and S160 that have been described as cold-sensitive [2, 33]. Both are old materials produced in the 1980s. In contrast with the cold-tolerant inbred line (S68911, class 1), the inbred line S160 showed a dominance of gene down-regulation over up-regulation under moderately cold conditions (approximately 15 °C) at the third leaf stage [33]. The line is further characterised by notably slow early growth at low field temperatures and rapid recovery at the onset of favourable thermal conditions (Prof. J. Adamczyk, Plant Breeding Smolice Co., Ltd., personal communication). Therefore, we hypothesised that although class 3 plants are sensitive to low temperatures, they could avoid the adverse effects of cold stress on the seedling transition to the autotrophic phase by arresting the VE stage. It remains to be determined the level of efficacy that this adaptation strategy could achieve under prolonged cold stress and/or soil crusting, both of which can have a significant effect on seedling emergence and overall plant growth.

We are aware that the low temperatures used in this study (day/night: 16/12 °C) resulted in only mild stress enabling even slow-growing inbred lines to reach the V1 growth stage of growth and develop a functional photosynthetic apparatus, limiting the general conclusions of the study to moderate cold stress only. Another limitation of our study is the small number of inbred lines studied, thereby hindering further generalisation of the results. Nonetheless, we believe that the finding of new, diverse strategies for enduring cold conditions during the early growth stages of maize warrants further extensive studies that include the investigation of physiological and molecular basis of new adaptation mechanisms, as well as the phenotype–genotype relationship.

## Conclusions

Automated plant phenotyping supported by the clustering of time series of morphological and spectral parameters and spectral indices confirmed the hypothesis of the diverse character of the response of maize seedlings to low temperatures at the early growth stages. We found two classes of inbred lines with clearly contrasting modes of both growth and changes in physiological status at low temperatures and during regrowth: class 4 contained cold-tolerant inbred lines, whereas class 3 combined cold-sensitive materials. We also found two classes that deviated from this simple and obvious division: class 1, which contained small seedlings with good physiological status, and class 2, which represented rapidly growing seedlings despite poor physiological status. The protective role of the xeromorphic body shape in maize seedlings under cold conditions has been earlier postulated [5–7]; however, the second mode of maize development at low temperatures has received little attention [7]. The underlying mechanism remains unknown and warrants further investigation because it may operate in European dent lines [7] and possibly in some elite US dent and even flint lines (this study). The developmental mode represented by class 3, i.e., the delay in the VE stage at low temperatures, is most intriguing and warrants further investigation because it may protect seedlings from cold injury during the transition to the autotrophic growth phase.

Future studies in this field should determine the physiological and molecular bases of the new promising mechanisms of maize adaptation to cold at early growth stages described in this report. Moreover, a more diverse set of phenotyped inbred lines should be used to identify additional genotypes belonging to the identified classes and/or new classes that may reveal other mechanisms of maize adaptation to cool spring conditions than those already described.

## Supplementary Information

Additional file 1. Maximal quantum yield of PSII (Fv/Fm) and photochemical reflection index (PRI) in the first, fully developed leaf (V1 growth stage) of plants of 64 inbred lines grown for 10–12 days at optimal temperatures (24/21°C; CONTROL) or for 19–22 days at low temperatures (16/12°C; COLD). Control and low-temperature values are marked with blue and orange bars, respectively. Measurements were performed at room temperature after 30 min dark acclimatisation. Experiments were repeated three times with three or four plants per experiment. Lines are sorted according to their Fv/Fm values for cold-grown plants. Data are presented as means ± SD marked as error bars. The emmeans function was used to conduct posthoc pairwise comparisons between all inbred lines—separately for each treatment condition. The Tukey’s method was used for P-value adjustment with ɑ = 5%. Different letters mark significantly different values. Results of statistical analysis is shown in Additional file 9. Grey and red bars regard inbred lines selected for Experiment II.

Additional file 2. Spectral indices of maize seedlings grown at low temperature till V1 growth stage (cold) and after 3-day-long recovery at optimal temperatures (regrowth). The lines are sorted according to their hue values for cold-grown plants. Experiments were repeated three to five times with five plants per experiment. The emmeans function was used to conduct posthoc pairwise comparisons between all inbred lines—separately for each treatment condition. The Tukey’s method was used for P-value adjustment with ɑ = 5%. Different letters mark significantly different values.

Additional file 3. Leaf hue and morphological traits of seedlings grown at low temperature till V1 growth stage (cold) and after 3-day-long recovery at optimal temperature (regrowth). Lines are sorted according to their hue values for cold-grown plants. Experiments were repeated three to five times with five plants per experiment. The emmeans function was used to conduct posthoc pairwise comparisons between all inbred lines—separately for each treatment condition. The Tukey’s method was used for P-value adjustment with ɑ = 5%. Different letters mark significantly different values.

Additional file 4. Averaged time series of the morphological parameters height, leaf area, leaf area index, and light penetration depth for the four classes in Figure 3. Data were subjected to a z-transformation, with mean and standard deviation calculated for all experimental data (data shown in Additional file 11).

Additional file 5. Averaged time series of spectral parameter greenness and the spectral vegetation index NPCI for the four classes in Figure 3. Data were subjected to a z-transformation, with mean and standard deviation calculated for all experimental data (data shown in Additional file 11).

Additional file 6. List of inbred lines used in Experiment I.

Additional file 7. List of inbred lines used in Experiment II.

Additional file 8. List of inbred lines used in Experiment III.

Additional file 9. Statistics to Additional file 1.

Additional file 10. Statistics to Fig. 2.

Additional file 11. The mean and standard deviation calculated for morphological parameters, spectral parameters, and spectral vegetation indices for all the experimental data.

## Data Availability

The phenotypic data were deposited in the data repository of the University of Warsaw (10.58132/UMADIT).
