# Supplementary material for: Diverse coping modes of maize in cool environment at early growth
Source: BMC Plant Biol. 2025 Feb 13;25:191. doi: 10.1186/s12870-025-06198-2 (PMC11823182; doi:10.1186/s12870-025-06198-2)
Supplement: Supplementary file 1 — Additional file 1. Maximal quantum yield of PSII (Fv/Fm) and photochemical reflection index (PRI) in the first, fully developed leaf (V1 growth stage) of plants of 64 inbred lines grown for 10–12 days at optimal temperatures (24/21°C; CONTROL) or for 19–22 days at low temperatures (16/12°C; COLD). Control and low-temperature values are marked with blue and orange bars, respectively. Measurements were performed at room temperature after 30 min dark acclimatisation. Experiments were repeated three times with three or four plants per experiment. Lines are sorted according to their Fv/Fm values for cold-grown plants. Data are presented as means ± SD marked as error bars. The emmeans function was used to conduct posthoc pairwise comparisons between all inbred lines—separately for each treatment condition. The Tukey’s method was used for P-value adjustment with ɑ = 5%. Different letters mark significantly different values. Results of statistical analysis is shown in Additional file 9. Grey and red bars regard inbred lines selected for Experiment II. [file 12870_2025_6198_MOESM1_ESM.docx]

| 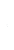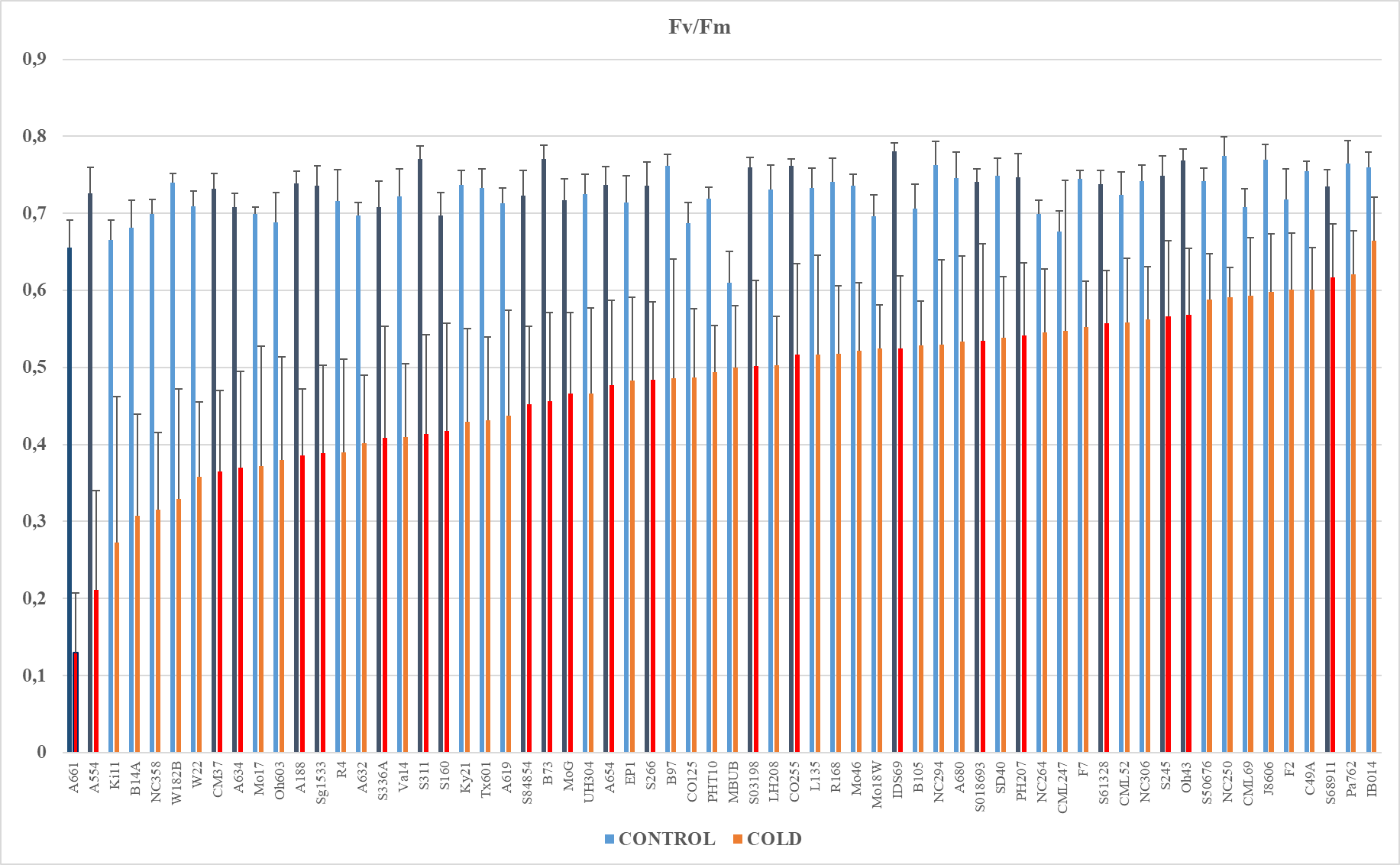 |
| --- |
| 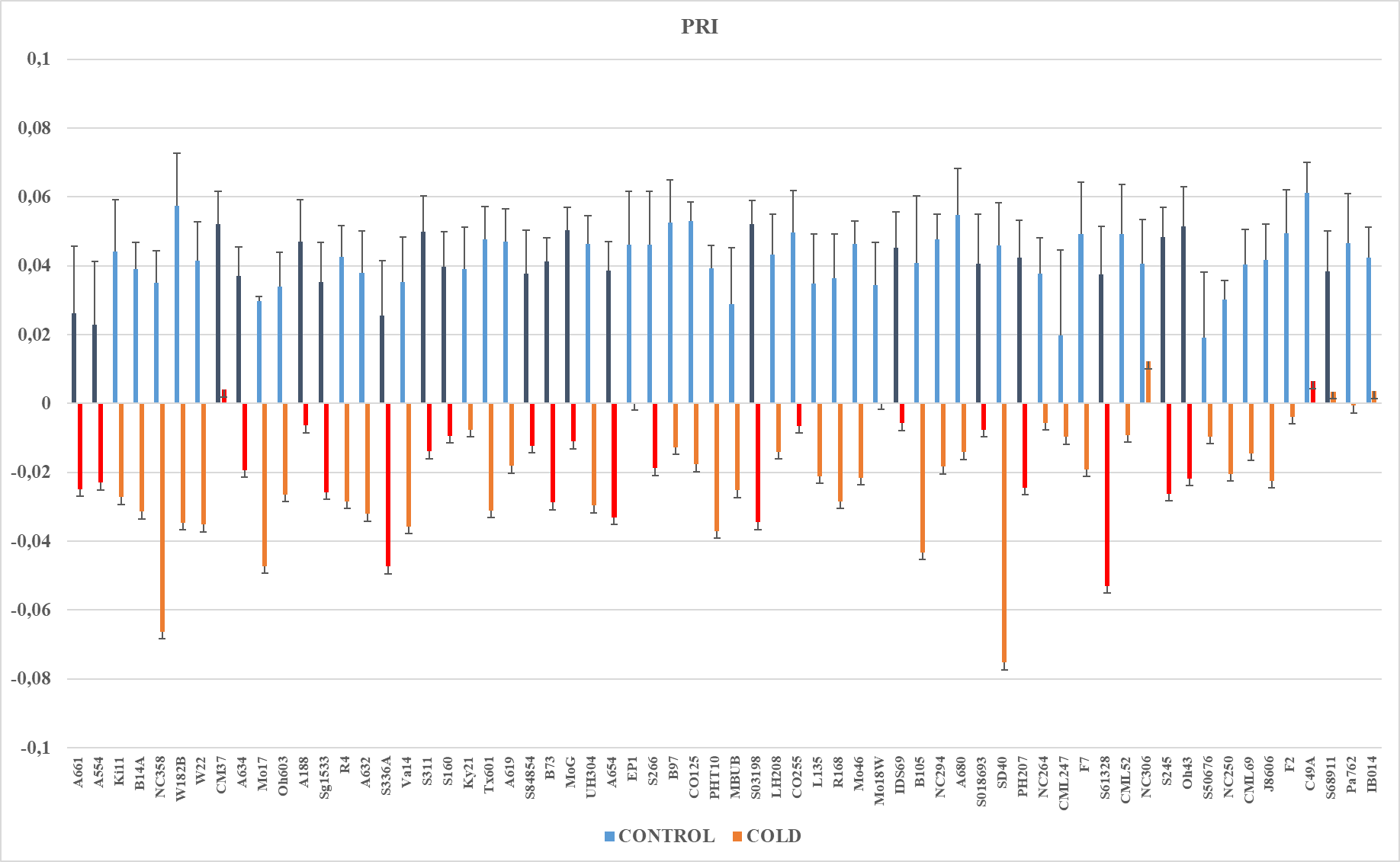 |

Additional file 1. Maximal quantum yield of PSII (Fv/Fm) and photochemical reflection index (PRI) in the first, fully developed leaf (V1 growth stage) . Measurements were performed on plants of 64 inbred lines grown at optimal temperatures (24°C/21°C; CONTROL) or at low temperatures (16°C/12°C; COLD). Experiments were repeated three times with three or four plants per experiment. Lines are sorted according to their Fv/Fm values for cold-grown plants. Control and low-temperature values are marked with blue and orange bars, respectively. Grey and red bars regard inbred lines selected for Experiment II.
