# Supplementary material for: Diverse coping modes of maize in cool environment at early growth
Source: BMC Plant Biol. 2025 Feb 13;25:191. doi: 10.1186/s12870-025-06198-2 (PMC11823182; doi:10.1186/s12870-025-06198-2)
Supplement: Supplementary file 3 — Additional file 3. Leaf hue and morphological traits of seedlings grown at low temperature till V1 growth stage (cold) and after 3-day-long recovery at optimal temperature (regrowth). Lines are sorted according to their hue values for cold-grown plants. Experiments were repeated three to five times with five plants per experiment. The emmeans function was used to conduct posthoc pairwise comparisons between all inbred lines—separately for each treatment condition. The Tukey’s method was used for P-value adjustment with ɑ = 5%. Different letters mark significantly different values. [file 12870_2025_6198_MOESM3_ESM.docx]

| HUE [°] | |
| --- | --- |
| 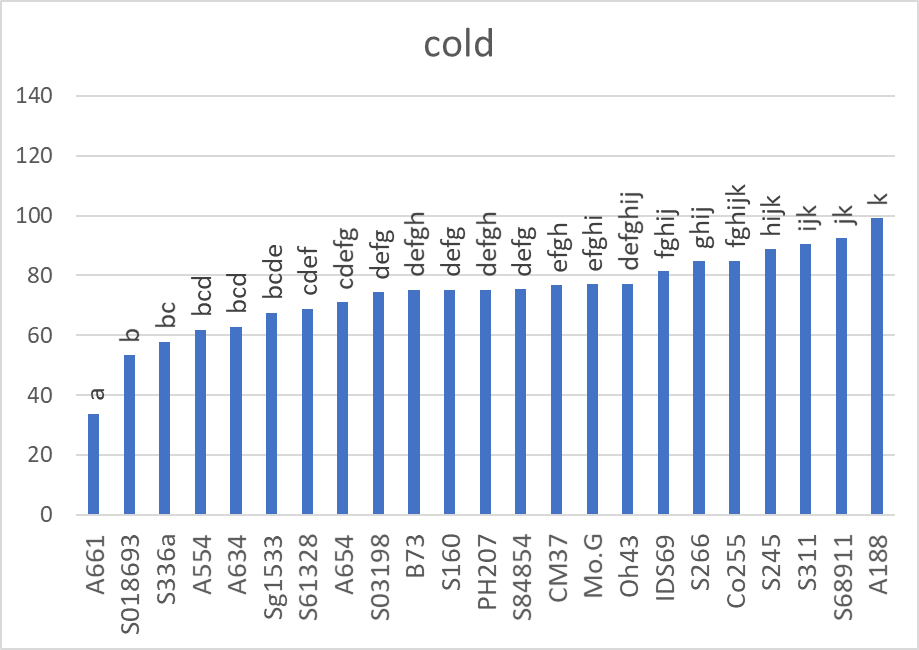 | 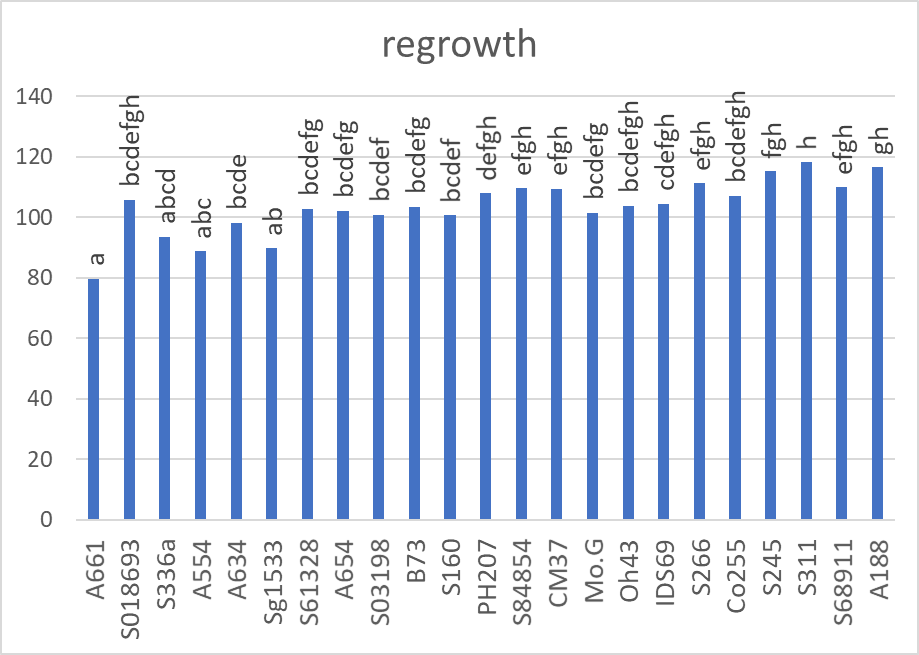 |
| Digital biomass [mm^3^] | |
| 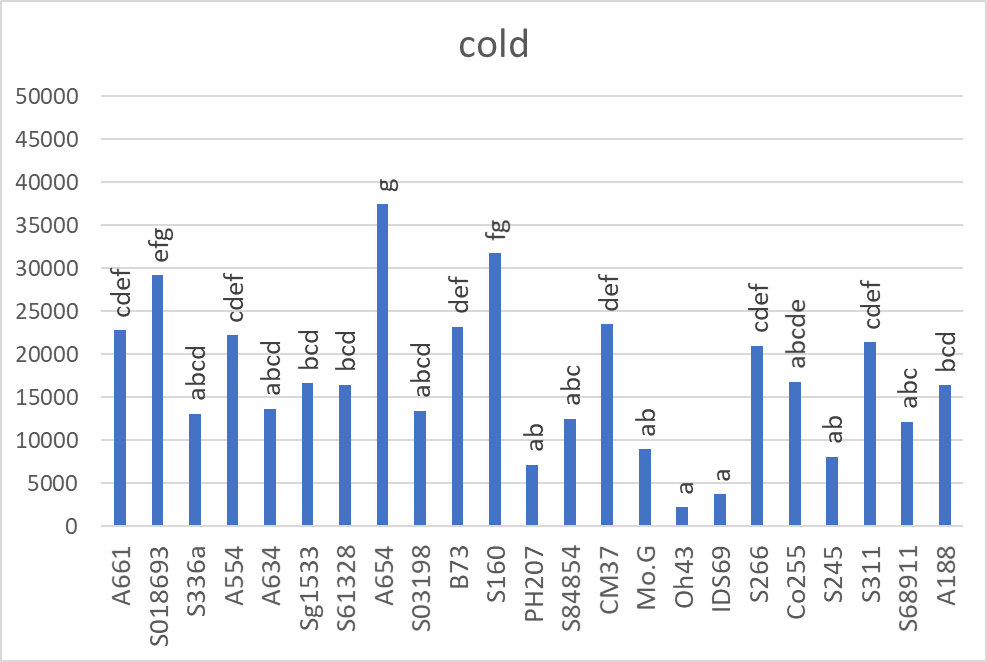 | 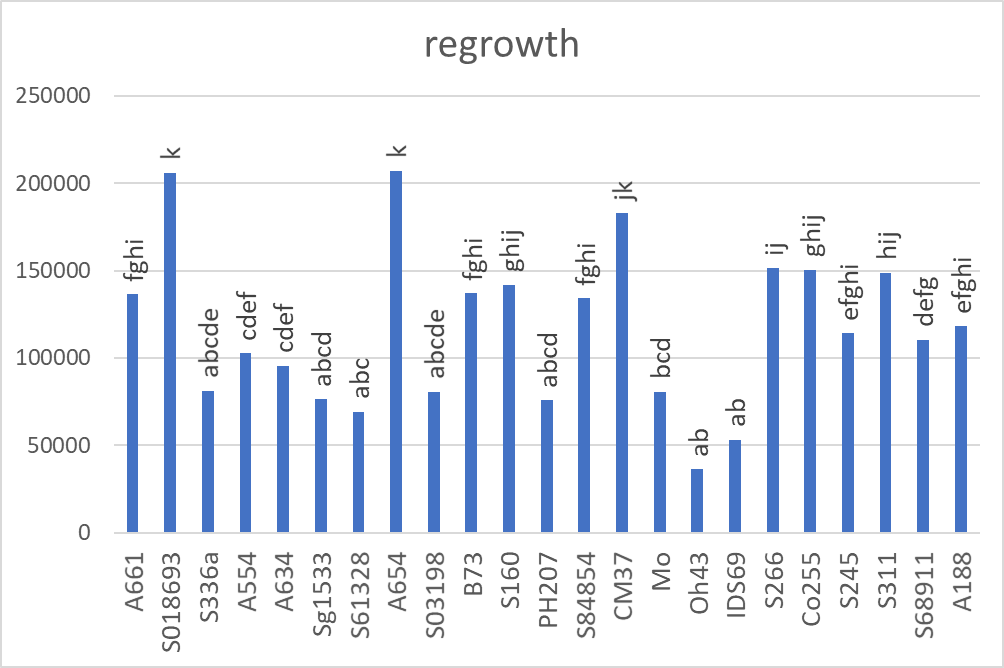 |
| Leaf area [mm^2^] | |
| 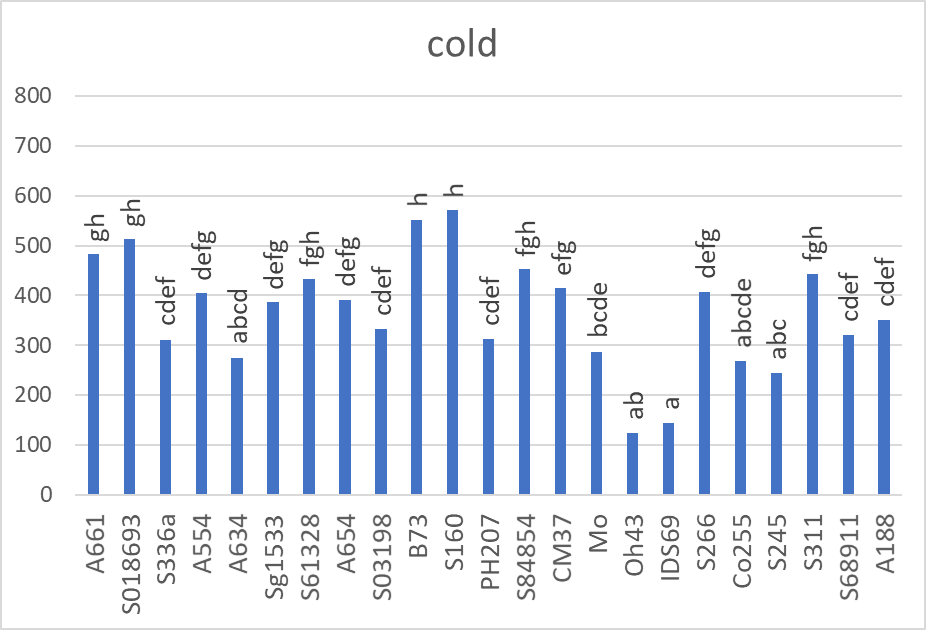 | 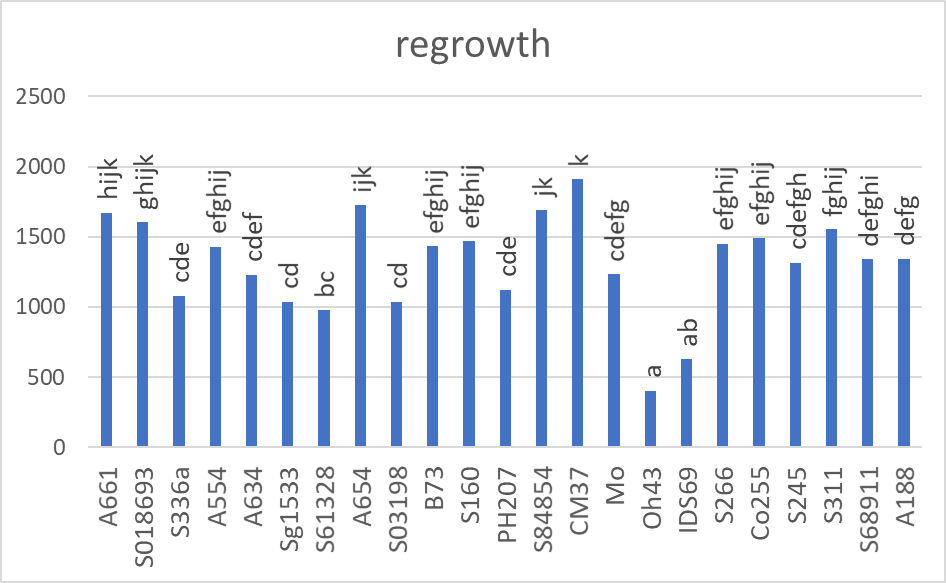 |

Additional file 3. Leaf hue and morphological traits of seedlings grown at low temperature till V1 growth stage (cold) and after 3-day-long recovery at optimal temperature (regrowth). The lines are sorted according to their HUE values for cold-grown plants. The experiments were repeated three to five times with five plants per experiment. The emmeans function was used to conduct post-hoc pairwise comparisons between all inbred lines, separately for each treatment condition. Tukey method was used for P-value adjustment with ɑ=5%. Different letters mark significantly different values.
