# Supplementary material for: Diverse coping modes of maize in cool environment at early growth
Source: BMC Plant Biol. 2025 Feb 13;25:191. doi: 10.1186/s12870-025-06198-2 (PMC11823182; doi:10.1186/s12870-025-06198-2)
Supplement: Supplementary file 4 — Additional file 4. Averaged time series of the morphological parameters height, leaf area, leaf area index, and light penetration depth for the four classes in Figure 3. Data were subjected to a z-transformation, with mean and standard deviation calculated for all experimental data (data shown in Additional file 11). [file 12870_2025_6198_MOESM4_ESM.docx]

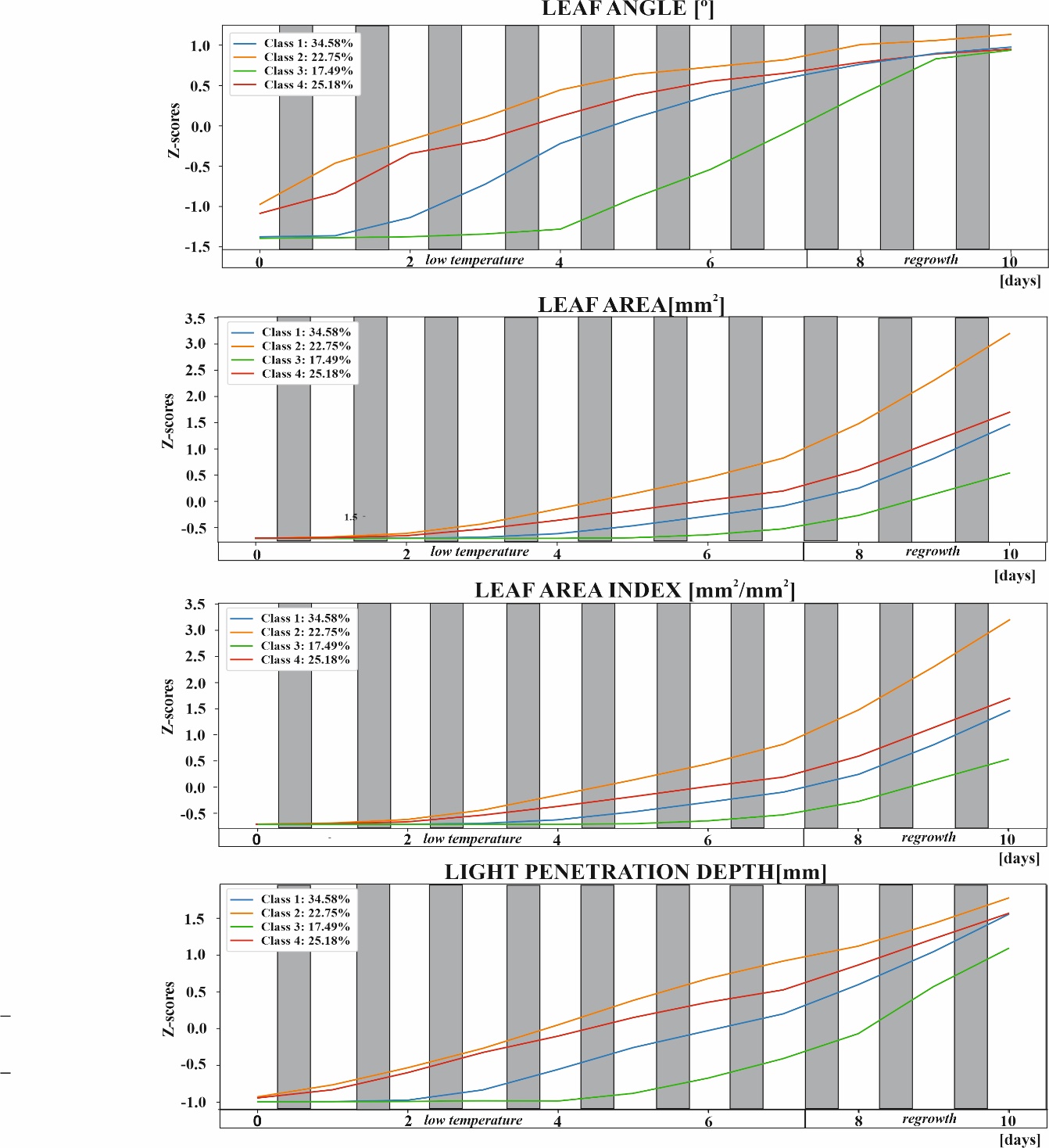


Additional file 4. Averaged time series of the morphological parameters height, leaf area, leaf area index, and light penetration depth for the four classes in Figure 3. Data were subjected to a z-transformation, with mean and standard deviation calculated for all experimental data (data shown in Table S3).
