# Supplementary material for: Diverse coping modes of maize in cool environment at early growth
Source: BMC Plant Biol. 2025 Feb 13;25:191. doi: 10.1186/s12870-025-06198-2 (PMC11823182; doi:10.1186/s12870-025-06198-2)
Supplement: Supplementary file 5 — Additional file 5. Averaged time series of spectral parameter greenness and the spectral vegetation index NPCI for the four classes in Figure 3. Data were subjected to a z-transformation, with mean and standard deviation calculated for all experimental data (data shown in Additional file 11). [file 12870_2025_6198_MOESM5_ESM.docx]

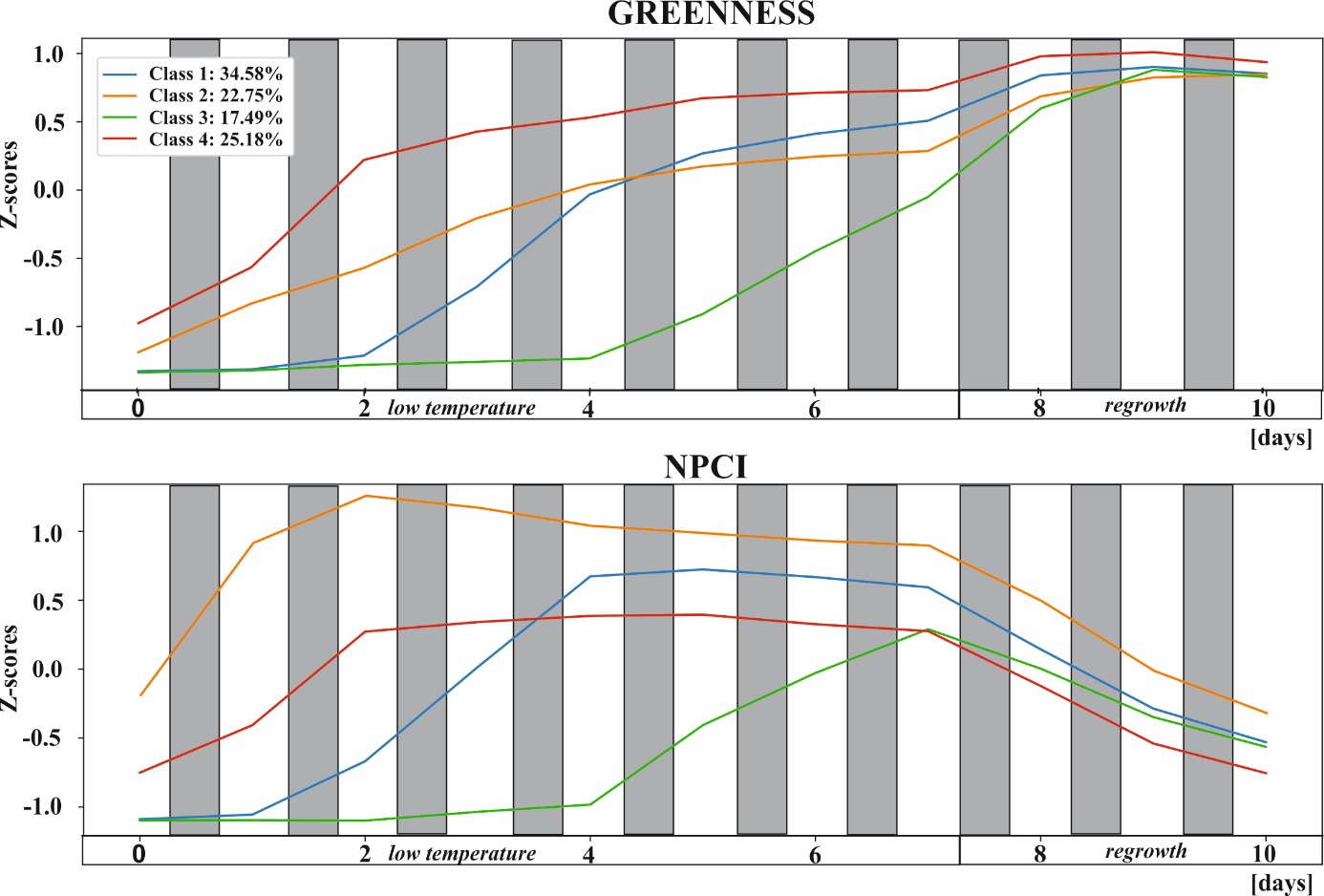


Additional file 5. Averaged time series of spectral parameter greenness and the spectral vegetation index NPCI for the four classes in Figure 3. Data were subjected to a z-transformation, with Mean and standard deviation calculated for all experimental data (data shown in Table S3).
