# Supplementary material for: Diverse coping modes of maize in cool environment at early growth
Source: BMC Plant Biol. 2025 Feb 13;25:191. doi: 10.1186/s12870-025-06198-2 (PMC11823182; doi:10.1186/s12870-025-06198-2)
Supplement: Supplementary file 6 — Additional file 6. List of inbred lines used in Experiment I. [file 12870_2025_6198_MOESM6_ESM.docx]

Additional file 6. A list of the inbred lines used in Experiment I

| No | Inbred line | Population group |  | No | Inbred line | Population group |
| --- | --- | --- | --- | --- | --- | --- |
| 1 | A188 | NSS |  | 33 | Mo46 | NSS |
| 2 | A554 | IO |  | 34 | MoG | NSS |
| 3 | A619 | NSS |  | 35 | NC250 | NSS |
| 4 | A632 | SS |  | 36 | NC264 | TS |
| 5 | A634 | SS |  | 37 | NC294 | SS |
| 6 | A654 | NSS |  | 38 | NC306 | SS |
| 7 | A661 | NSS |  | 39 | NC358 | TS |
| 8 | A680 | SS |  | 40 | Oh43 | NSS |
| 9 | B105 | MIX |  | 41 | Oh603 | MIX |
| 10 | B14A | SS |  | 42 | Pa762 | NSS |
| 11 | B73 | SS |  | 43 | PH207 | IO |
| 12 | B97 | NSS |  | 44 | PHT10 | SS |
| 13 | C49A | NSS |  | 45 | R168 | NSS |
| 14 | CM37 | NSS |  | 46 | R4 | NSS |
| 15 | CML247 | TS |  | 47 | S018693 | MIX |
| 16 | CML52 | TS |  | 48 | S03198 | IO |
| 17 | CML69 | TS |  | 49 | S160 | NSS |
| 18 | CO125 | NSS |  | 50 | S245 | MIX |
| 19 | CO255 | MIX |  | 51 | S266 | NSS |
| 20 | EP1 | MIX |  | 52 | S311 | NSS |
| 21 | F2 | MIX |  | 53 | S336A | SS |
| 22 | F7 | MIX |  | 54 | S50676 | IO |
| 23 | IB014 | IO |  | 55 | S61328 | MIX |
| 24 | IDS69 | NSS |  | 56 | S68911 | IO |
| 25 | J8606 | IO |  | 57 | S84854 | IO |
| 26 | Ki11 | TS |  | 58 | SD40 | MIX |
| 27 | Ky21 | NSS |  | 59 | Sg1533 | NSS |
| 27 | L135 | IO |  | 60 | Tx601 | TS |
| 29 | LH208 | SS |  | 61 | UH304 | IO |
| 30 | MBUB | NSS |  | 62 | Va14 | NSS |
| 31 | Mo17 | NSS |  | 63 | W182B | NSS |
| 32 | Mo18W | TS |  | 64 | W22 | NSS |

Abbreviations

IO - Iodent

MIX – Mixed

NSS – Non-stick stalk

SS – Stick stalk

TS - Tropical/subtropical (TS) germplasm
