# Supplementary material for: Diverse coping modes of maize in cool environment at early growth
Source: BMC Plant Biol. 2025 Feb 13;25:191. doi: 10.1186/s12870-025-06198-2 (PMC11823182; doi:10.1186/s12870-025-06198-2)
Supplement: Supplementary file 8 — Additional file 8. List of inbred lines used in Experiment III. [file 12870_2025_6198_MOESM8_ESM.docx]

Additional file 8. A list of the inbred lines used in Experiment III

| No | Inbred line | Population group | Country of origin |
| --- | --- | --- | --- |
| 1 | A188 | NSS | USA |
| 2 | A554 | NSS | USA |
| 3 | A634 | SS | USA |
| 4 | A654 | NSS | USA |
| 5 | A661 | NSS | USA |
| 6 | B73 | SS | USA |
| 7 | B97 | NSS | USA |
| 8 | C49A | NSS | USA |
| 9 | CM37 | NSS | Canada |
| 10 | Co255 | MIX | Canada |
| 11 | D06 | MIX | Germany |
| 12 | D152 | MIX | Germany |
| 13 | IDS69 | NSS | USA |
| 14 | Mo46 | NSS | USA |
| 15 | MoG | Iodent | USA |
| 16 | Oh43 | NSS | USA |
| 17 | R168 | NSS | USA |
| 18 | S018693 | MIX | Poland |
| 19 | S03198 | Iodent | Poland |
| 20 | S160 | CGP-Dent | Poland |
| 21 | S245 | Iodent/SS | Poland |
| 22 | S266 | F2/EP1 | Poland |
| 23 | S311 | Lancaster/Co255 | Poland |
| 24 | S336a | Iodent/SS | Poland |
| 25 | S68911 | SS/Iodent | Poland |
| 26 | S84854 | Iodent | Poland |
| 27 | Sg1533 | NSS | USA |
| 28 | UH304 | Iodent | Germany |
| 29 | W182B | NSS | USA |
| 30 | W22 | NSS | USA |

Abbreviations

CGP – Canadian gene pool

IO - Iodent

Lancaster – Lancaster germplasm

MIX – Mixed

NSS – Non-stick stalk

SS – Stick stalk

Co255, EP1, F2 – elite inbred lines
