# Supplementary material for: Diverse coping modes of maize in cool environment at early growth
Source: BMC Plant Biol. 2025 Feb 13;25:191. doi: 10.1186/s12870-025-06198-2 (PMC11823182; doi:10.1186/s12870-025-06198-2)
Supplement: Supplementary file 11 — Additional file 11. The mean and standard deviation calculated for morphological parameters, spectral parameters, and spectral vegetation indices for all the experimental data. [file 12870_2025_6198_MOESM11_ESM.docx]

Additional file 11. The mean and standard deviation calculated for morphological parameters, spectral parameters, and spectral vegetation indices for all the experimental data

Digital biomass [mm³]

Mean: 21097.941884180527

SD: 38850.60808931434

Height Max [mm]

Mean: 33.812968123383186

SD: 36.30237388360789

Height [mm]

Mean: 32.17507773167176

SD: 29.225025989683054

Leaf area [mm²]

Mean: 325.36279793310837

SD: 460.49346992774736

NDVI average []

Mean: 0.1764079039565086

SD: 0.20585097013825496

NPCI average []

Mean: 0.1949155737791026

SD: 0.1760772534575984

PSRI average []

Mean: 0.3488449393983165

SD: 0.6072869662460526

HUE average [°]

Mean: 59.869518444184735

SD: 41.21052295134427

Greenness average []

Mean: 0.14886545500505563

SD: 0.11027950811551837

Leaf angle [°]

Mean: 22.02387176473678

SD: 15.671837422044577

Leaf area (projected) [mm²]

Mean: 191.02756821792968

SD: 292.900384950026

Leaf area index [mm²/mm²]

Mean: 0.022822324793507806

SD: 0.03232608783073021

Leaf inclination [mm²/mm²]

Mean: 1.6819796076320042

SD: 1.3091398408997652

Light penetration depth [mm]

Mean: 18.39251812141347

SD: 18.495309448128342
